# The P0.1 maneuver as an alternative method for assessing the validity of esophageal pressure measurements during assisted ventilation: an exploratory analysis

**DOI:** 10.1186/s40635-026-00873-w

**Published:** 2026-03-01

**Authors:** Tatiana M. Bastian, Christine Eimer, Friederike Behmüller, Norbert Weiler, Giacomo Bellani, Dirk Schädler, Tobias Becher

**Affiliations:** 1 Department of Anesthesiology and Operative Intensive Care Medicine, University Medical Center Schleswig-Holstein, Campus Kiel, Kiel, Germany; 2 Centre for Medical Sciences - CISMed, University of Trento, Trento, Italy; 3 Department of Anesthesia and Intensive Care, Santa Chiara Hospital, Trento, Italy

**Keywords:** Assisted mechanical ventilation, Transpulmonary pressure, Esophageal pressure, P0.1

## Abstract

**Background:**

Transpulmonary pressure, calculated as the difference between airway pressure (Paw) and esophageal pressure (Pes), is an important monitoring parameter during assisted mechanical ventilation, provided Pes is measured via a correctly placed and filled esophageal pressure probe. The reference method to verify Pes accuracy in spontaneously breathing patients requires calculating the ratio of changes in Pes and Paw (ΔPes/ΔPaw) during an inspiratory effort against an occluded airway. We hypothesized that the P0.1 maneuver, a brief and repeatable test, could provide an alternative means to assess ΔPes/ΔPaw during assisted mechanical ventilation.

**Methods:**

We performed an exploratory secondary analysis of data from a multicenter prospective observational study (ICEBERG study; NCT05203536). In 35 patients receiving assisted mechanical ventilation, ΔPes/ΔPaw obtained during P0.1 maneuvers (Ratio_P0.1_, experimental method) was compared with ΔPes/ΔPaw from prolonged expiratory occlusion maneuvers (Ratio_occ_, reference method) using linear regression and Bland–Altman analysis.

**Results:**

Among 25 patients with 65 evaluable measurements, Ratio_P0.1_ showed a moderate correlation (*R*^2^:0.647, *p* < 0.0001) with Ratio_occ_. Bland–Altman analysis demonstrated minimal bias and acceptable agreement between methods. Using the occlusion maneuver as reference, Ratio_P0.1_ identified incorrect Pes measurement with a sensitivity of 93% and a specificity for identifying correct Pes measurement of 71%. Results were consistent across patient subgroups.

**Conclusions:**

Our exploratory analysis suggests that the P0.1 maneuver may support semi-continuous screening of esophageal pressure signal validity during assisted ventilation. While abnormal P0.1 values should prompt confirmatory occlusion testing, values within the expected range may help rule out major measurement errors. These findings provide a rationale for prospective validation studies including different ventilator types.

*Trial registration*: clinicaltrials.gov, NCT05203536. Registered 24. January 2022—Retrospectively registered, https://classic.clinicaltrials.gov/ct2/show/NCT05203536

## Background

Despite being a central element of intensive care for patients with acute respiratory failure, mechanical ventilation can also contribute to further lung damage. Traditional ventilation parameters such as tidal volumes and airway pressures, although crucial, do not adequately reflect the mechanical stress imposed on lung tissue [1, 2] even more so in the presence of spontaneous breathing efforts. To assess and minimize mechanical stress within the framework of a lung-protective ventilation strategy, it is necessary to determine the difference between airway pressure and pleural pressure. Due to the anatomical proximity of the pleura to the esophagus, esophageal pressure (Pes) can serve as a surrogate for pleural pressure [3], provided it is obtained via a correctly placed and filled esophageal pressure probe.

In patients undergoing assisted mechanical ventilation with preserved spontaneous breathing activity, confirming the correct position and blocking of the esophageal pressure probe typically involves an end-expiratory airway occlusion during a full inspiration, which produces a change in pleural pressure. Synchronous amplitude changes in esophageal and airway pressure (∆Pes and ∆Paw) during the occlusion maneuver indicate correct position and adequate blocking of the esophageal balloon [4, 5]. While effective, this method has its drawbacks: it permits only point measurements at specific times. It is not easily automated and may be uncomfortable for the patient due to the extended airway occlusion. These limitations underscore the need for a continuous, automated monitoring system to ensure the validity of esophageal pressure measurements during assisted mechanical ventilation.

The P0.1 maneuver presents a possible solution to some of these issues. It is frequently used in ventilated patients with preserved spontaneous breathing activity to assess central respiratory drive as a predictor of weaning success [6, 7]. In the P0.1 maneuver, the patient inhales against a closed valve at the end of expiration, but unlike the expiratory occlusion maneuver, this is kept for 100 ms only, a duration hardly perceived by the patient. Furthermore, the P0.1 maneuver can be easily repeated in an automated fashion. However, whether this brief measurement sequence is sufficient to evaluate correct esophageal probe position and blocking remains uncertain. This study aims to analyze data obtained in the framework for the ICEBERG study to establish proof-of-concept for the feasibility of using the P0.1 maneuver as an alternative method to determine the correct placement and blocking of esophageal pressure probes in patients undergoing assisted mechanical ventilation.

## Methods

This is an exploratory, hypothesis-generating secondary analysis of prospectively collected data from the ICEBERG observational study (NCT05203536). Ethical approval was granted by the local ethics committee (D573/21). This study analyzed the impact of mechanical system properties on outcomes during assisted ventilation in patients with acute hypoxemic respiratory failure. A total of 35 patients were included. Exclusion criteria comprised age below 18 years, pregnancy, moribund patient status, high bronchopleural fistula volume, severe chronic obstructive pulmonary disease with home oxygen therapy or home ventilation, and patients who had already undergone a successful spontaneous breathing trial.

Ventilation was administered using the Elisa 800 VIT intensive care ventilator (Löwenstein Medical, Bad Ems, Germany). Only patients who had received esophageal pressure catheters (NutriVent; Sidam, Mirandola, Italy) for routine clinical monitoring were included in this analysis. To pursue our hypothesis, we integrated esophageal pressure (Pes) measurement with ventilator data, including airway pressure (Paw), airflow (⩒), and volume (V) recorded at a rate of 200 Hz (Hz) and exported for further analysis. Data processing was performed using Microsoft Excel, and data visualization and comparison were carried out using GraphPad Prism (Graphpad, Boston, USA).

**Fig. 1:**
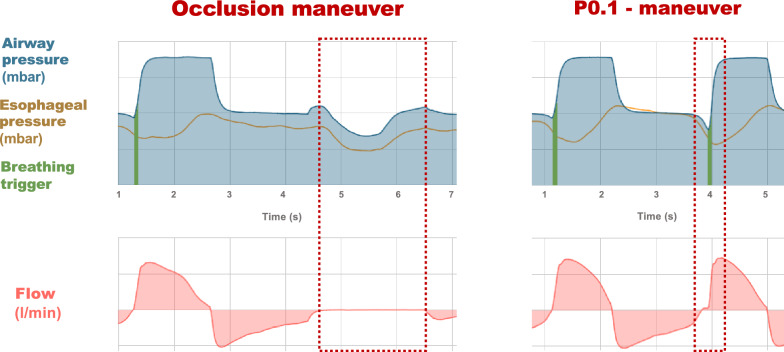
Principle of occlusion and P0.1 maneuver measurements. Representative airway pressure, esophageal pressure, and flow signals recorded during an expiratory occlusion maneuver and a P0.1 maneuver in the same patient

Three P0.1 maneuvers per occlusion maneuver were recorded for each patient (Fig. 1). P0.1 maneuvers were recorded prior to occlusion maneuvers without verification of the esophageal catheter’s position and blocking. Occlusion maneuvers were recorded with various blocking volumes to ensure optimal placement and blocking of the catheter. To ensure comparability between maneuvers, we included only those measurements in which end-expiratory Pes, measured 0.5 s before the onset of inspiration, deviated by no more than 20% between the P0.1 and occlusion maneuvers, indicating a similar blocking volume and catheter position between maneuvers.

The ratio between changes in esophageal pressure and airway pressure (ΔPes/ΔPaw) was calculated both for expiratory occlusion maneuvers (Ratio_occ_ = (ΔPesΔPaw)_occ_, the reference method) and P0.1 maneuvers (Ratio_P0.1_ = (ΔPes/ΔPaw)_P0.1_, the experimental method). Subsequently, the slopes of the ΔPes/ΔPaw curves were compared for both maneuvers. P0.1 maneuvers yielding physiologically implausible negative values of Ratio_P0.1_ as well as maneuvers with P0.1 > −1 mbar, indicating insufficient respiratory drive, were removed from the analysis.

A correct probe position was defined as follows:

For the occlusion maneuver: 0.75 < Ratio_occ_ < 1.25,

For the P0.1 maneuver: 0.75 < Ratio_P0.1_ < 1.25.

The correlation between Ratio_occ_ and Ratio_P0.1_ was analyzed by linear regression. Agreement between both methods was analyzed by the Bland–Altman method. To account for repeat measurements within the same patient, we further assessed the association between Ratio_occ_ and Ratio_P0.1_ using a Gaussian GEE with identity link, clustering by patient and using an exchangeable working correlation; robust standard errors were used. Correct or incorrect positioning and blocking of the esophageal pressure probe were determined using the occlusion maneuver—the established standard—as ground truth. Sensitivity and specificity were calculated according to the ability of the P0.1 maneuver to correctly identify a correct or incorrect catheter positioning/blocking. Fisher’s exact test was used to determine statistical significance.

## Results

**Fig. 2:**
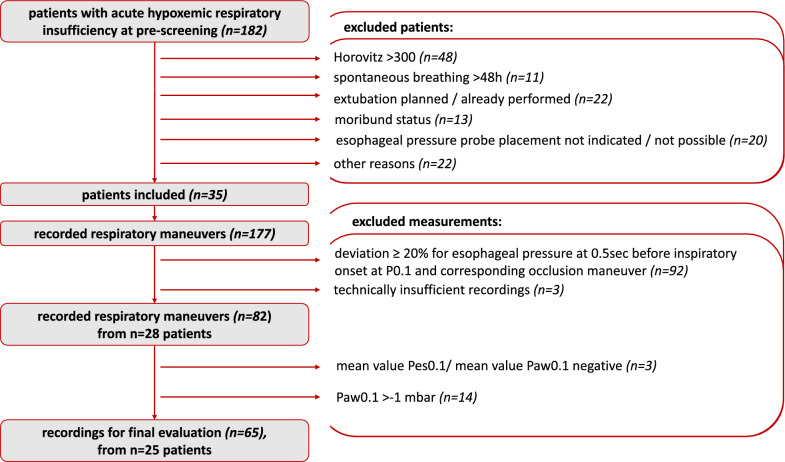
Patient selection. Flow diagram showing patient inclusion and selection of evaluable measurements for analysis

Three Ratio_P0.1_ values were negative and were removed from the analysis as physiologically implausible. After excluding breaths with insufficient respiratory drive, defined as P0.1 > −1 mbar, we obtained a final dataset of 65 evaluable measurements from 25 patients (Fig. 2). Characteristics of these patients included a median age of 65 years, with varying primary indications for ICU admission, comorbidities, and durations of controlled and assisted ventilation (Table 1).

**Table 1:** Clinical characteristics of *n* = 25 patients included in the final analysis

Clinical characteristics	Median (range) or *n*/25 (%)
Age	65 years (36–85 years)
Sex	Female: 9/25 (36%) / male: 16/25 (64%)
Body mass index	28 kg/m^2^ (21–43 kg/m^2^)
Primary respiratory failure at ICU admission	6/25 (24%)
Pulmonary comorbidities	5/25 (20%)
Cardiovascular comorbidities	17/25 (68%)
SOFA score at ICU admission	11 (5–18)
RASS score at ICU admission	−4 (−5 to 0)
Horovitz quotient at ICU admission	194 (121–285)
Indications for ICU admission	ARDS/pneumogenic sepsis: 5/25 (20%) Non-pneumogenic sepsis: 4/25 (16%) Major vascular surgery: 4/25 (16%) Neurologic disease: 3/25 (12%) Polytrauma: 3/25 (12%) Cardiopulmonary resuscitation: 2/25 (8%) Other: *n* = 4/25 (16%)
Tracheotomy	18/25 (72%)
Controlled ventilation	4 days (1–21 days in total)
Assisted ventilation	7 days (3–18 days in total)
Hospitalization	29 days (7–70 days in total)
ICU length of stay	23 days (7–40 days in total)
Discharge from ICU	18/25 (72%)
Maneuver measurements	3 (1–6 per patient)

*n* = 25 patients (65 measurements) remained for the final correlation analysis after excluding implausible/low-drive maneuvers. Continuous variables are represented as medians with range

**Fig. 3:**
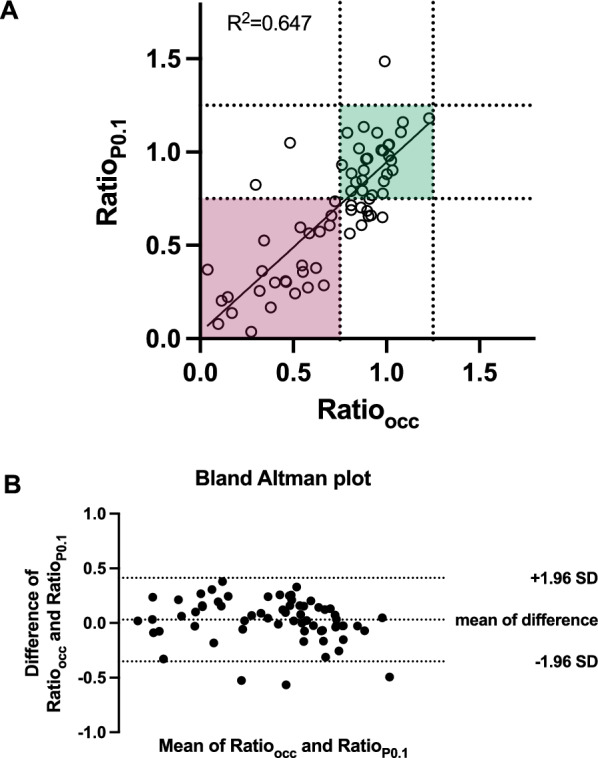
Correlation of ΔPes/ΔPaw during occlusion and P0.1 maneuver measurements. **A** Linear regression analysis of Ratio_P0.1_ and Ratio_occ_ (*n* = 65 measurements from *n* = 25 patients). The solid line represents the regression fit; dotted lines indicate the predefined range for acceptable probe positioning and blocking (0.75–1.25). **B** Bland–Altman plot showing agreement between Ratio_P0.1_ and Ratio_occ_. The solid line indicates mean bias, and dashed lines represent the 95% limits of agreement

Linear regression analysis revealed a moderate correlation between Ratio_occ_ and Ratio_P0.1_ (*R*^2^ = 0.647, *p* < 0.0001; Fig. 3A). This correlation was independent of the patient’s clinical baseline characteristics based on comparison of independent linear regressions [8] according to the patient’s age, sex, body mass index, or ICU admission for primary respiratory failure vs. secondary respiratory complications and presence or absence of pre-existing pulmonary comorbidities (Table 2). Bland–Altman analysis showed a mean bias of 0.03 (95% CI −0.018 to 0.079) with 95% limits of agreement of − 0.352 to 0.412. Three measurements lay outside these limits together confirming an agreement between P0.1 and occlusion maneuver (Fig. 3B). A cluster-adjusted GEE confirmed a moderate-to-good association between Ratio_occ_ and Ratio_P0.1_ (*β* = 0.886, 95% CI 0.740–1.031), consistent with the unadjusted regression (*R*^2^ ≈ 0.65) indicating that repeated measurements within the same patient did not introduce systematic biases.

**Table 2:** Correlation between Ratio_P0.1_ and Ratio_occ_ in relation to patient baseline variables

Feature	*R*^2^ Group 1	*R*^2^ Group 2
Age	< Median	> Median
	0.550	0.700
Sex	Male	Female
	0.685	0.608
BMI	< Median	> Median
	0.679	0.635
Primary respiratory failure	Yes	No
	0.871	0.634
Pulmonary comorbidities	Yes	No
	0.833	0.583

Out of 38 correct probe positions/blockings identified by the occlusion maneuver (Ratio_occ_), 27 were also correctly identified by the P0.1 maneuver (Ratio_P0.1_). Conversely, the P0.1 maneuver correctly identified 25 out of 27 incorrect probe positions/blockings indicated by the occlusion maneuver. This resulted in a sensitivity for detecting incorrect Pes signal transduction of 93% and a specificity of 71% when compared to the occlusion maneuver. In our cohort, the positive predictive value of Ratio_P0.1_ for identifying a patient with incorrect Pes measurements was 69%, and the negative predictive value (i.e., the likelihood of finding correct Pes measurements in patients with Ratio_P0.1_ within the expected range) was 93%.

## Discussion

The primary objective of this study was to explore whether the P0.1 maneuver could serve as a screening tool for assessing esophageal probe positioning and blocking during assisted ventilation, in comparison with the occlusion maneuver. Our findings indicate a moderate correlation between these two methods, suggesting that the P0.1 maneuver could effectively support semi‑continuous screening of esophageal balloon validity during assisted ventilation, to complement—rather than replace—the standard occlusion test.

A correct catheter placement/blocking as identified by the P0.1 maneuver (Ratio_P0.1_) was confirmed by the occlusion maneuver (Ratio_occ_) in 27 of 29 measurements, resulting in a negative predictive value of 93%. This indicates that a complete occlusion maneuver could frequently be omitted when Ratio_P0.1_ is within the expected range (0.75–1.25). However, in 11 out of 38 measurements with correct placement/filling of the catheter, the P0.1 maneuver wrongly indicated a catheter misplacement/misblocking, resulting in a relatively poor positive predictive value (69%). This increased false-positivity rate is probably related to the shortness of the P0.1 maneuver. Here, cardiac oscillations in particular might negatively influence the measurement and lead to false-positive results. Leveraging Fourier transformations [9, 10] to filter for such interfering signals might help to further increase the method’s specificity. In this context, the high negative predictive value is particularly relevant, as it supports the use of P0.1 as a screening or rule-out tool, whereas the lower positive predictive value reinforces the need for confirmatory occlusion testing when abnormal values are detected. One may assume that, after an initial “formal” evaluation of correct position, the semi-automated technique could continuously monitor and alarm if malposition/deflation occurred. Indeed, in an automated setting, repeated P0.1 measurements at short time intervals might be able to exclude such technical variation before an alarm is filed.

It is essential to acknowledge the limitations of this study. First, this was an exploratory secondary analysis not designed for formal method validation. Second, the final sample size was relatively small, comprising 25 patients and 65 evaluable measurements, which limits statistical power and generalizability. Nonetheless, these patients represented real-world cases from a mixed intensive care unit, encompassing a diverse range of respiratory failure etiologies. Third, all measurements were obtained using a single ventilator platform, and performance characteristics of the P0.1 maneuver may differ across devices. Fourth, the P0.1 technique is likely to be suitable only for patients with sufficient respiratory drive undergoing assisted mechanical ventilation. We defined P0.1 < -1 mbar as the cutoff for adequate central respiratory drive and respiratory effort, with implausible measurements being obtained in patients with lower respiratory drive. In patients with insufficient respiratory drive or in patients on mechanical ventilation, the occlusion technique in combination with external chest compressions remains the standard, although it may be associated with patient discomfort due to the required chest compressions.

Validation of the P0.1 maneuver as test required a substantial number of measurements in clinical situations where the esophageal probe was insufficiently placed or filled. We performed P0.1 maneuvers without previous checking on the probe position and blocking. Occlusion maneuvers were recorded with various blocking volumes to ensure optimal placement and blocking of the catheter. To include only comparable measurements in the final evaluation, we required the end-expiratory Pes to have less than 20% deviation between P0.1 and occlusion maneuver 0.5 s before the maneuver. This cutoff resulted in the removal of a substantial proportion of maneuvers from analysis. However, this strategy enabled us to analyze a hypothesis-generating dataset obtained during real-world situations without artificially inducing catheter misplacement/misblocking.

Although the occlusion maneuver is widely accepted as the clinical reference standard for validating esophageal pressure measurements, it is not without limitations. Its accuracy depends on adequate patient effort, stable respiratory mechanics, and proper timing of the maneuver, and it provides only intermittent point assessments rather than continuous validation.

Automated P0.1 maneuvers have been implemented into many intensive care ventilators, providing an automated function for monitoring patients' respiratory drive during weaning [11]. However, performance characteristics for the P0.1 trigger phase might vary between ventilators. Based on our hypothesis-generating dataset obtained using the Elisa 800 VIT intensive care ventilator, our observations provide a rationale for a future prospective validation study including different ventilator types.

In the future, an automated analysis of Ratio_P0.1_ during automatically performed P0.1 maneuvers could be useful for semi-continuous monitoring of esophageal balloon positioning and potentially for adjusting esophageal balloon blocking volumes automatically. Initiated with a standard occlusion maneuver for calibration, automated P0.1 maneuvers could be performed at 1–5 min intervals to ensure continuous monitoring of correct catheter placement/blocking using the P0.1 maneuver as a rule-out or screening approach, whereas the occlusion maneuver remains necessary for confirmation when P0.1-based measurements fall outside the expected range. This hypothesis-generating study lays the groundwork for such automation, demonstrating that the P0.1 maneuver may be suitable for confirming correct esophageal probe placement and blockings across diverse clinical scenarios and patient characteristics.

## Data Availability

Raw data will be made available from the corresponding author upon reasonable request.

## Abbreviations

Pes: Esophageal pressure
Paw: Airway pressure
ΔPes/ΔPaw: Change in Pes and Paw
Ratio_occ_: ΔPes/ΔPaw obtained during expiratory occlusion maneuver
Ratio_P0.1_: ΔPes/ΔPaw obtained during P0.1 maneuver
BMI: Body mass index
